# Comparison of Quantitative Evaluation and Conventional Scar Scale Analysis for Pediatric Pathological Scars

**DOI:** 10.3390/biomedicines14040784

**Published:** 2026-03-30

**Authors:** Jin-Ye Guan, Xing Zou, Jun-Wen Ge, Rui-Cheng Tian, Wei Liu, Mei-Yun Li, Dan Deng

**Affiliations:** 1 Department of Dermatology, Shanghai Children’s Medical Center, Shanghai Jiao Tong University School of Medicine, Shanghai 200127, China; gjy120505@163.com (J.-Y.G.); zouxing930525@163.com (X.Z.); gejw0917@163.com (J.-W.G.); tianruicheng@scmc.com.cn (R.-C.T.); 2 Shanghai Key Laboratory of Tissue Engineering, Department of Plastic and Reconstructive Surgery, Shanghai Ninth People’s Hospital, Shanghai Jiao Tong University School of Medicine, Shanghai 200011, China; liuwei_2000@yahoo.com

**Keywords:** pediatric pathological scars (PPS), Antera 3D^®^, VSS, dermoscopy

## Abstract

**Background/Objectives**: The incidence of pediatric pathological scars (PPS) has been gradually increasing due to various causes, highlighting the need for accurate scar assessment to monitor disease progression and therapeutic efficacy. Vancouver Scar Scale (VSS) and other scar evaluation systems are relatively subjective evaluation methods that rely on physicians’ or patients’ own judgment. By contrast, when comparing different scar scale evaluation methods, a three-dimensional (3D) camera and dermoscopy may provide relatively objective measurable parameters to avoid possible subjective bias created by the observers. This study aimed to compare the utility of traditional VSS evaluation with that of 3D cameras and dermoscopy in PPS evaluation. **Methods**: A total of 35 pediatric patients (aged 0–18 years) with PPS were involved, and their scars were assessed via the VSS, dermoscopy, and the Antera 3D^®^ system. In addition, a subset of 18 patients (36 scar regions) was also evaluated for therapeutic efficacy after 3–6 months of treatment. Briefly, VSS scores were blindly evaluated by two independent dermatologists under standardized conditions. Quantitative assessment was also performed using dermoscopy and the Antera 3D^®^ system. The former quantified chromatic parameters (pigmentation: L*, vascularity: a*, green value); the latter captured multispectral 3D images to analyze volume, pigmentation, and erythema. Data are presented as means ± standard deviation and analyzed using paired-sample *t* tests (one-tailed), the Wilcoxon signed-rank test, and standardized response means (SRMs) to assess therapeutic sensitivity, while baseline variability was evaluated using the standard deviation and coefficient of variation (CV). **Results:** The results showed that Antera 3D^®^ detected significant reductions in pigmentation (*p* < 0.01, SRM = −0.46), vascularity (*p* < 0.001, SRM = −0.59), and volume (*p* < 0.0001, SRM = −0.83), while dermoscopy indicated similar moderate improvements in vascularity (Green value: *p* < 0.001, SRM = 0.57; a*: *p* < 0.0001, SRM = −0.68) and pigmentation (L*: *p* < 0.0001, SRM = 0.48) after treatments. VSS showed significant gains in pliability (*p* < 0.0001, SRM = −1.13), height (*p* < 0.01, SRM = −0.54), and overall impression (*p* < 0.0001, SRM = −0.86), but minimal changes in pigmentation (*p* > 0.05, SRM = 0) or vascularity (*p* > 0.05, SRM = −0.21). At baseline, Antera 3D^®^ showed the greatest variability in pigmentation (CV 43.41%) and volume (CV 91.21%), followed by VSS in vascularity (CV 52.95%), pliability (CV 34.05%), and overall impression (CV 31.76%). Dermoscopy presented the lowest variability, indicating limited discriminative power. **Conclusions:** In conclusion, Antera 3D^®^ offers an objective, sensitive, and spatially precise approach for PPS assessment and may provide additional quantitative information for evaluating subtle and early changes alongside traditional scar assessment scales. Its integration into clinical practice will enhance treatment monitoring and support more accurate timing of therapeutic interventions.

## 1. Introduction

Scars are the result of the healing process. The ideal scar is thin, flat, pale, soft, elastic, and painless. Pathological scarring results from dysregulation of the healing process, especially during the modeling stage. This leads to long-term inflammation and excessive cell proliferation and collagen production, called hypertrophic or keloid scarring [1,2]. The most severe pathological scar is a keloid, which significantly affects appearance, as well as physical and mental health, and may even develop into a suppurative keloid [3,4]. The incidence of scarring in children aged 0–18 years has increased steadily, with approximately 1 million new cases diagnosed annually in developed countries [5]. Pathological scars cause varying degrees of discomfort in children and may lead to a series of complications, including pain, pruritus, abnormal tactile sensation, and even severe dysfunction [6,7]. In addition to physiological effects, scars can also lead to psychological issues such as depression, anxiety, and even suicidal tendencies [8,9]. Severe scars impose significant economic and social burdens on families and society.

Various therapeutic approaches for pathological scars in children have been reported in the literature. These options include massage, pressure therapy, silicone sheets, topical or intralesional corticosteroids, cryotherapy, surgery, radiation (X-rays, radium, and iridium), antineoplastic agents, and laser therapy [1,10]. However, to date, no single method has been proven superior to others. Scars undergo a prolonged maturation process, during which different treatments must be applied, particularly for children whose scarring progression is unpredictable. A recent study revealed that the timing and interval of treatment can significantly influence the therapeutic outcomes of PPS [11]. To tailor treatments, it is essential to assess various parameters associated with scar formation [12]. Currently, the primary methods for scar assessment include various scar assessment scales: the Vancouver Scar Scale, modified Vancouver Scar Scale, Seattle scale, Mapping Assessment of Scars and Photographs, Hamilton Scale, Manchester Scale, Patient and Observer Scar Assessment Scale, Stony Brook Scar Evaluation Scale, University of North Carolina “4P” scale, Visual Analog Scale, and Dermatology Life Quality Index [13,14]. Conventional scar assessment methods rely largely on subjective evaluations with limited incorporation of objective and quantifiable parameters. Therefore, both subjective and objective assessment approaches are essential for accurately evaluating scar severity.

The Antera 3D^®^ system was developed by Miravex to overcome the limitations of subjectivity and lack of detail in the current evaluation methods. It provides objective measurements of skin texture and color, enabling quantitative evaluation of various skin conditions. Recently, several studies have evaluated scars using Antera 3D^®^ cameras to provide valuable parameters for monitoring scar progression and evaluating treatment efficacy. For scar therapeutic evaluation, Antera 3D^®^ is objective and accurate and thus worthy of wide promotion [15,16,17]. However, there are still several limitations in the reported studies. Most research in this field has been conducted in the general population, with a limited focus on pediatric patients. This represents a significant research gap, as children account for approximately 70% of individuals with hypertrophic scars and face unique clinical challenges, including rapid growth, hormonal fluctuations, and limited capacity for accurate self-assessment. These developmental characteristics make objective scar evaluation particularly crucial in this vulnerable population [5]. Current clinical practice mainly relies on conventional scar assessment scales, such as the Vancouver Scar Scale (VSS). This study focused on pediatric pathological scars to compare evaluation sensitivity between VSS and quantitative assessments of the Antera 3D^®^ system and dermoscopy in order to provide a precise and objective assessment of PPS.

## 2. Materials and Methods

### 2.1. Study Design

The present work was a longitudinal exploratory study approved by the Institutional Review Board (IRB) of Shanghai Children’s Medical Center, affiliated with the Shanghai Jiao Tong University School of Medicine (IRB ID: SCMCIRB-K2024171-1). The study was conducted in accordance with the principles of the 1964 Declaration of Helsinki and its subsequent amendments. Scars were assessed using a dermoscopy (FotoFinder, Bad Birnbach, Germany) and a three-dimensional imaging system (Antera 3D^®^, Miravex Ltd., Dublin, Ireland).

### 2.2. Participants

Patients were recruited from the Dermatology Department of Shanghai Children’s Medical Center between September 2023 and November 2025. Participants and their parents were thoroughly informed of the study details and signed an informed consent prior to assessment. Participants were free to withdraw from the study at any time without consequence.

The inclusion criteria were as follows: (i) children aged 0–18 years diagnosed with pathological scars; (ii) patients with scars longer than 3 months and less than 2 years; (iii) patients with scars suitable for standardized imaging and objective assessment.

The exclusion criteria were as follows: (i) patients older than 18 years; (ii) scars located in areas with irregular contours, such as the toes or fingers; (iii) patients with systemic or local conditions that could interfere with scar healing or assessment, including immunosuppression, diabetes, lactation, or local/general infection; (iv) patients who had received previous scar treatments (e.g., surgery, laser, or corticosteroid injection); (v) patients with connective tissue disorders or hypersensitivity to lidocaine; (vi) patients scheduled for scar resection.

### 2.3. Study Process

Background information of all eligible patients was collected, including age, sex, cause of injury, and days post-injury. The causes of injury included trauma and surgery. Days post-injury represented the duration from the time of injury to enrollment.

**Figure 1:**
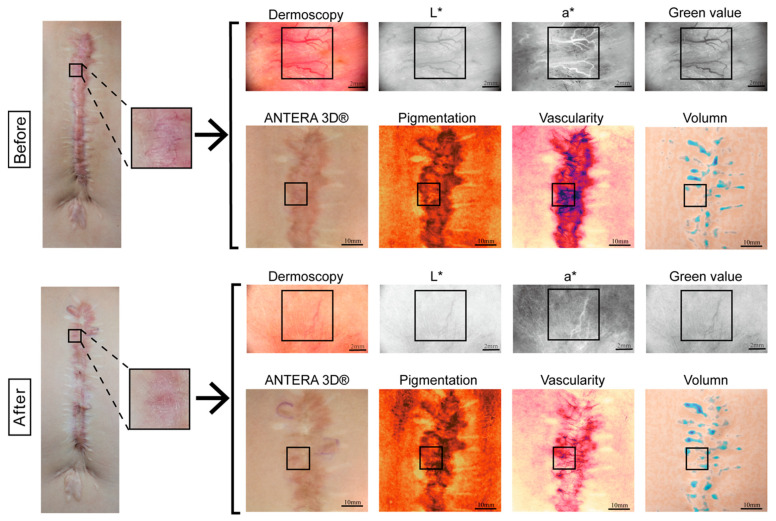
Representative workflow of scar selection and imaging analysis. (VSS: Pigmentation, Vascularity, Height, Pliability; Dermoscopy: Green value, a* value, L* value; Antera 3D^®^: Pigmentation, Vascularity, Volume). Images in the first and third rows: Scale bar = 2 mm. Images in the second and fourth rows: Scale bar = 10 mm.

At baseline, 35 pediatric patients with PPS were recruited. Each patient underwent scar assessment using the Vancouver Scar Scale (VSS), dermoscopy, and the Antera 3D^®^ system to evaluate overall scar features. See Figure 1.

For longitudinal evaluation of treatment response, a subset of 18 patients was followed up after receiving scar therapy. A total of 36 scar regions (10 mm × 10 mm each) were delineated and assessed before and after treatment in these patients. For imaging analysis, scars requiring treatment were preferentially selected, particularly those presenting with prominent pigmentation, vascularity, or elevation. Generally, the selected areas also need to reflect the diverse characteristics of the different regions so as to avoid induced clustering of data from the same patient. Measurements were primarily obtained from representative regions within the central area of the scar, which were considered to best reflect typical scar characteristics while avoiding the boundary between scar tissue and surrounding normal skin. The follow-up duration ranged from 3 to 6 months. Some patients contributed more than one scar region, reflecting heterogeneity within large scar areas.

All scars were evaluated by two independent assessors, each with at least two years of clinical experience in scar management. Dermoscopic measurements were performed by one assessor who was blinded to the results of the VSS and the Antera 3D^®^ system. To minimize environmental bias, all assessments were conducted in the same room under consistent lighting and temperature.

### 2.4. Measurements

#### 2.4.1. VSS

The VSS is a widely used clinical tool designed to evaluate the characteristics of scars, particularly hypertrophic scars and keloids. The scale includes four parameters—vascularity, pigmentation, height, and pliability—each scored from 0 to 3, with higher total scores indicating increased scar severity.

#### 2.4.2. Dermoscopy

Dermoscopic images of the scars were analyzed using ImageJ (version 1.54p). For each image, a 6 mm × 6 mm square region of interest (ROI) was uniformly selected to ensure consistency across samples. The images were converted into the CIELAB color space, and two key parameters were extracted: the L* value (lightness) and the a* value (red–green axis). In addition, the green channel intensity was calculated separately from the RGB color space to evaluate vascular-related chromatic changes [18]. The a* value and green value reflect the degree of vascularity, whereas the L* value reflects the level of pigmentation within the scar. All the images were preprocessed using the rolling-ball algorithm for background subtraction, noise filtering, and contrast enhancement before parameter extraction to ensure accuracy and reproducibility.

#### 2.4.3. Antera 3D^®^

The Antera 3D^®^ system is a portable imaging device for skin imaging and analysis that uses LEDs of multiple wavelengths to map chromophores such as melanin and hemoglobin. Unlike traditional RGB imaging, it employs seven wavelengths for precise colorimetric analysis. When connected to a laptop, it captures images in less than 1 s and generates reports in approximately 90 s. The software allows manipulation of the Antera 3D^®^ system, measures keloid volume, and assesses color, melanin, and hemoglobin density before and after treatment, noting the distribution and uniformity within scars.

### 2.5. Data Analysis

In this study, we used four normality tests (the Shapiro–Wilk, Kolmogorov–Smirnov, D’Agostino–Pearson, and Anderson–Darling tests) to assess the normality of the scar evaluation indices. If any test yielded a *p* < 0.05 for an index, its data were deemed normally distributed.

Descriptive data, including sex, age group, and cause of injury, were described in terms of frequency and percentage, whereas age and days post-injury were described in terms of the mean and standard deviation.

For the longitudinal treatment–response analysis, paired-sample *t* tests (one-tailed) were conducted to compare pre- and post-treatment values obtained by the three measurement tools (VSS, dermoscopy, and Antera 3D^®^), and the Wilcoxon signed-rank test was additionally performed for VSS to provide a nonparametric assessment. Statistical significance was defined as *p* < 0.05. To further evaluate the responsiveness of each tool, the standardized response mean (SRM) was calculated as the mean change divided by the standard deviation of the change. An SRM ≥ 0.80 was considered large, 0.50–0.79 was considered moderate, and 0.20–0.49 was considered small. Tools with higher SRM values were interpreted as indicating greater responsiveness to treatment-induced changes.

For the baseline analysis, interindividual variability was quantified for each method by calculating the standard deviation (SD) and coefficient of variation (CV = SD/mean). Greater baseline variability was interpreted as indicating greater sensitivity of the measurement tool in discriminating PPS heterogeneity across patients.

## 3. Results

### 3.1. Demographics and Injury-Related Details

Thirty-five patients were enrolled in this study. As shown in Table 1, 20 (57.15%) female participants and 15 (42.85%) male participants were included, with a mean age of 7.93 ± 3.59 years (range 0.75–18.00). With respect to etiology, 8 (25.86%) patients had scars resulting from trauma, and 27 (77.14%) patients had scars resulting from surgery. The locations of the lesions were predominantly in the trunk (13, 37.14%), followed by the head/face/neck (9, 25.71%) and the leg (6, 17.14%). Scars on the hand (5, 14.29%) and arm (2, 5.71%) were relatively uncommon. The mean duration of the scars was 5.94 months, and 28.57% of the patients had a follow-up of more than three months.

### 3.2. Comparison of Pre- and Post-Treatment VSS Scores and Antera 3D^®^ Data

A total of 36 scars from 18 patients who had received standardized treatment for more than 1 month were selected, and the changes in the VSS score, dermoscopy score, and Antera 3D^®^ score were analyzed before and after treatment. See Table 2.

**Figure 2:**
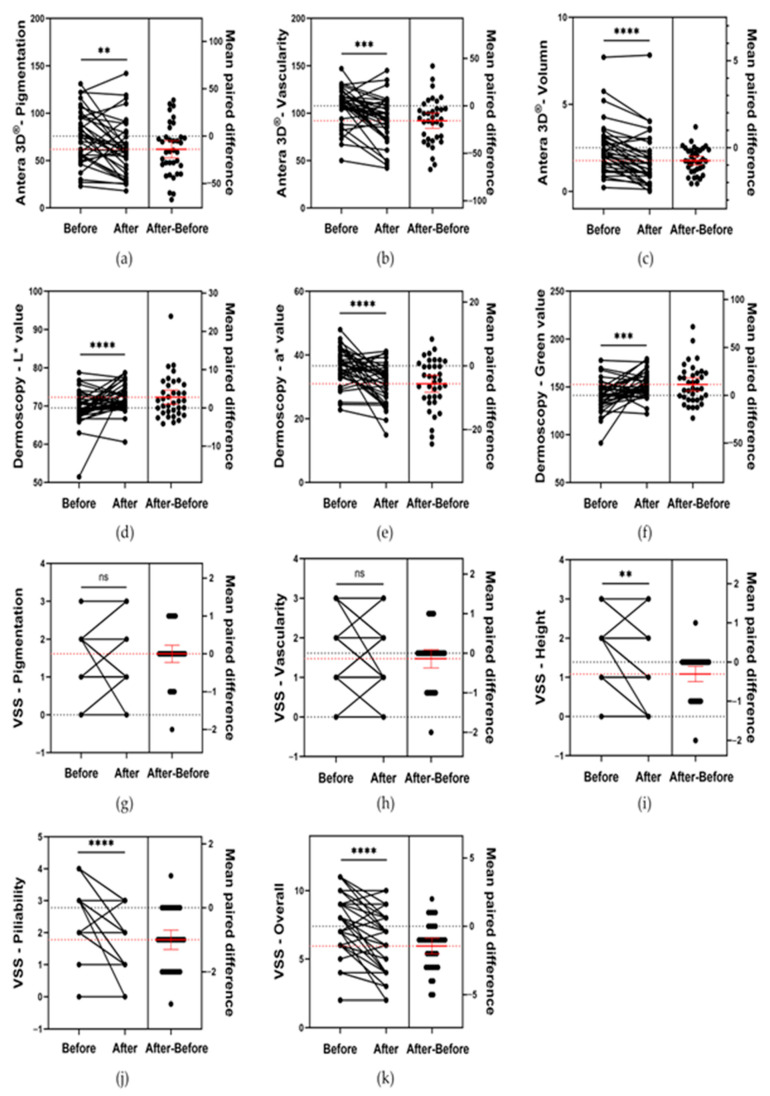
Estimation plots of paired *t* tests for pre-post changes in scar parameters assessed by VSS, dermoscopy, and Antera 3D^®^ for 36 scars from 18 patients. The red line and error bars indicate the mean ± SD of the paired differences. ns = *p* > 0.05; ** = *p* < 0.01; *** = *p* < 0.001; **** = *p* < 0.0001. (**a**–**c**): According to Antera 3D^®^ measurements, the analysis results of pigmentation, vascularity and scar volume. (**d**–**f**): According to Dermoscopy analysis, the analysis results of L*, a* and green value. (**g**–**k**): According to the VSS scores, the analysis results of pigmentation, vascularity, scar height, pliability and overall impression.

According to the VSS scores, no significant changes in pigmentation or vascularity were observed between pre- and post-treatment assessments (*p* > 0.05). However, scar height moderately decreased from 1.39 ± 0.79 to 1.08 ± 1.76 (*p* < 0.01), and both the pliability and overall impression scores significantly decreased (pliability, from 2.78 ± 0.85 to 1.78 ± 0.89 (*p* < 0.0001); overall, from 7.39 ± 2.10 to 5.94 ± 2.20 (*p* < 0.0001)). See Figure 2g–k. Similar results were obtained using Wilcoxon signed-rank tests. See Table S1.

Dermoscopy analysis revealed a significant decrease in the a* value (from 36.60 ± 5.74 to 30.98 ± 6.17 (*p* < 0.0001)) and a moderate increase in the green value (from 141.20 ± 15.94 to 152.53 ± 13.31 (*p* < 0.001)), indicating a moderate reduction in scar vascularity. In addition, the L* value moderately increased (from 69.50 ± 4.54 to 72.29 ± 3.72 (*p* < 0.0001)), suggesting an improvement in the pigmentation load. See Figure 2d–f.

Antera 3D^®^ measurements revealed moderate reductions in pigmentation (from 75.75 ± 27.72 to 61.89 ± 29.02 (*p* < 0.01)) and vascularity (from 107.69 ± 19.55 to 92.00 ± 24.20 (*p* < 0.001)) and a significant decrease in scar volume (from 2.59 ± 1.46 to 1.90 ± 1.58 (*p* < 0.0001)). See Figure 2a–c.

### 3.3. Baseline Variability Analysis of Scar Assessments in VSS, Dermoscopy, and Antera 3D^®^

Across all 35 patients, variability analysis revealed distinct performance among the three assessment tools. For pigmentation and volume, Antera 3D^®^ demonstrated greater variability (69.91 ± 30.35, CV = 43.41% for pigmentation; 15.87 ± 14.48, CV = 91.21% for volume), indicating higher sensitivity in distinguishing interindividual differences.

For vascularity, the VSS showed the highest variability (1.60 ± 0.85, CV = 52.95%), and it was the only tool that could assess pliability (2.37 ± 0.81, CV = 91.21%) and overall impressions (7.46 ± 2.37, CV = 31.76%), both of which have relatively high variability.

**Figure 3:**
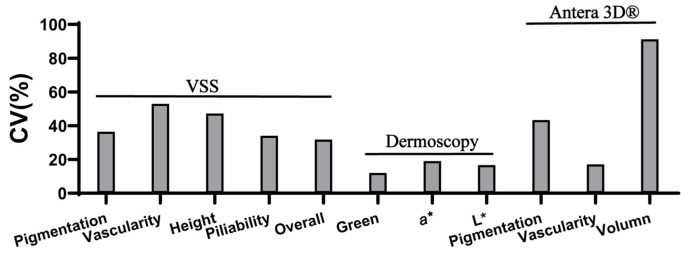
Baseline coefficient of variation (CV) values of scar parameters measured by VSS, dermoscopy, and Antera 3D^®^.

In contrast, the dermoscopic parameters exhibited lower variability for both vascularity (a* value: 35.58 ± 6.79, CV = 19.09%) and pigmentation (L* value: 68.63 ± 11.45, CV = 16.68%), suggesting more limited discriminative power in the baseline evaluation. See Table 3 and Figure 3.

## 4. Discussion

The incidence of PPS is steadily increasing. However, their evaluation still relies largely on subjective scales, which lack sufficient accuracy in capturing the dynamic and rapidly evolving characteristics of scars in children. Although objective assessment tools have been developed and are increasingly applied in scar evaluation, relatively few studies have specifically focused on pediatric populations. The Antera 3D^®^ system, a skin imaging system based on multispectral imaging and three-dimensional reconstruction technology, enables quantitative assessment of key scar parameters, including volume, pigmentation (melanin), and erythema (hemoglobin). Its advantages include being non-invasive, multiparametric, and reproducible, and several studies have highlighted its value in scar assessment. This study aimed to investigate the utility of a novel objective tool, Antera 3D^®^, in pediatric scar evaluation. Our findings demonstrated that Antera 3D^®^ was more sensitive in detecting pre- and post-treatment changes, including pigmentation, erythema, and volume changes. These results are consistent with those of Ruccia et al., who reported that Antera 3D^®^ reliably quantified changes in scar pigmentation, erythema, and volume before and after treatment [16]. Meanwhile, conventional scar assessment tools each possess distinct strengths. Dermoscopy demonstrates high sensitivity and low variability in the evaluation of vascular and pigmentary alterations, while the VSS remains widely applied for its sensitivity in overall scar assessment and its clinical convenience. By providing objective three-dimensional and volumetric parameters, Antera 3D^®^ complements these traditional assessment methods and enables a more comprehensive evaluation of pediatric scars.

Importantly, our study further contributes to the existing literature by highlighting the sensitivity of Antera 3D^®^ to detect interindividual variability, an aspect that has not been previously explored in pediatric scar evaluation. In this study, the coefficient of variation (CV) at baseline was used to quantify variability among patients. Assuming measurement error is adequately controlled, a higher CV reflects greater interindividual heterogeneity captured by the measurement tool, thereby indicating higher sensitivity in discriminating individual differences. Similar approaches have been applied in biological and clinical measurement studies, where CV is commonly used as a proxy for trait variability and discriminative ability [19,20]. The present study demonstrated that the Antera 3D^®^ system had superior sensitivity in detecting individual differences in pigmentation and volume compared with both VSS and dermoscopy in these parameters. Taken together, these findings suggest that the Antera 3D^®^ may serve as a valuable complementary tool for pediatric scar assessment. Notably, our study revealed that VSS demonstrated greater sensitivity to inter-individual variability in erythema compared to the other two tools. Given that erythema is a critical parameter in scar assessment and can serve as an indicator of scar maturation [21], our findings support the combined use of subjective and objective assessment methods in the evaluation of PPS.

Nevertheless, certain limitations of Antera 3D^®^ should be noticed. Firstly, due to its reliance on a 56 mm × 56 mm planar image acquisition field, its application is restricted to localized assessment of larger scars, making it difficult to capture an entire lesion, particularly when the scar spans multiple anatomical planes. Secondly, Antera 3D^®^ does not assess parameters such as pliability or subjective symptoms, which still require additional objective tools or subjective scales. In addition, the comparison among assessment tools in this study was mainly based on *p* values from paired tests and SRM values. Although these indicators reflect responsiveness to treatment-induced changes, they may not fully capture the overall performance of different assessment methods. Future studies incorporating larger cohorts and more comprehensive validation approaches are needed to further evaluate the sensitivity and reliability of scar assessment tools. Besides, using the CV values as an indicator of the sensitivity of scar assessment tools also has certain limitations. CV values may be influenced by multiple factors beyond the intrinsic characteristics of the scars themselves, including measurement stability and sample heterogeneity. Therefore, further studies are required to more rigorously evaluate the performance of different assessment methods. In terms of study design, limitations include the relatively small sample size and heterogeneity of treatment modalities, as patients underwent different interventions, potentially introducing variability.

Future studies should include larger and more diverse patient cohorts to further validate the performance of different scar assessment tools. In addition, longitudinal studies with standardized imaging protocols and multicenter collaboration may help reduce measurement variability and improve the comparability of results. Integrating objective imaging techniques with clinical evaluation scales may also provide a more comprehensive framework for scar assessment. Moreover, longitudinal data collection at multiple time points would allow for a more comprehensive investigation of the sensitivity of Antera 3D^®^ in monitoring sequential treatment outcomes in PPS. Ultimately, such efforts may contribute to the establishment of a more reliable reference standard for scar evaluation, particularly in pediatric populations where standardized assessment methods remain limited.

## 5. Conclusions

In summary, this study demonstrated that the Antera 3D^®^ provides a more comprehensive assessment of PPS, detecting interindividual differences in pigmentation, erythema, and volume with greater sensitivity for individual variability in volume and pigmentation. Although less detailed, VSS effectively identified interindividual differences in erythema, highlighting the continued clinical relevance of subjective scales. Conversely, dermoscopy demonstrated a limited ability to discriminate individual differences, underscoring the need for careful interpretation and combined use with more objective modalities. Taken together, these findings support the integration of 3D imaging into pediatric scar management as a reliable, non-invasive, and quantitative tool, with complementary roles for VSS and dermoscopy in comprehensive scar evaluation.

## Figures and Tables

**Table 1:** Baseline characteristics.

Characteristics	Participants (*n* = 35)
Sex: male, n (%)	15 (42.85%)
Mean age at injury	7.93 ± 3.59 (range 0.75–17.00)
Age at injury, n (%)	
Infant (birth-23 months)	1 (2.86%)
Preschool Child (2–5 years)	3 (8.57%)
Child (6–12 years)	24 (68.57%)
Adolescent (13–18 years)	7 (20.00%)
Etiology, n (%)	
Post-trauma	8 (25.86%)
Surgical	27 (77.14%)
Duration of visit, n (%)	
<3 month	25 (71.43%)
≥3 month	10 (28.57%)
Mean duration of visit (month)	5.94 ± 4.95
Scar location, n (%)	
Head/face/neck	9 (25.71%)
Trunk	13 (37.14%)
Arm	2 (5.71%)
Hand	5 (14.29%)
Leg	6 (17.14%)

Data are presented as mean ± standard deviation (range) or number (%).

**Table 2:** Pre-post differences (means ± SDs) and standardized response means (SRMs) of scar assessments by VSS, dermoscopy, and Antera 3D^®^ for 36 scars from 18 patients.

	Pre (Mean ± SD)	Post (Mean ± SD)	ΔMean ± SD (Post–Pre)	*p* Value	Effect Size (SRM)
VSS					
Pigmentation	1.61 ± 0.68	1.61 ± 0.76	0.00 ± 0.67	>0.05	0 (negligible)
Vascularity	1.61 ± 0.86	1.47 ± 0.76	−0.14 ± 0.67	>0.05	−0.21 (negligible)
Height	1.39 ± 0.79	1.08 ± 0.76	−0.31 ± 0.57	<0.01	−0.54 (moderate)
Pliability	2.78 ± 0.85	1.78 ± 0.89	−1.00 ± 0.88	<0.0001	−1.13 (large)
Overall	7.39 ± 2.10	5.94 ± 2.20	−1.44 ± 1.67	<0.0001	−0.86 (large)
Dermoscopy					
Green value	141.20 ± 15.94	152.53 ± 13.31	11.34 ± 19.96	<0.001	0.57 (moderate)
a*	36.60 ± 5.74	30.98 ± 6.17	−5.71 ± 8.37	<0.0001	−0.68 (moderate)
L*	69.50 ± 4.54	72.29 ± 3.72	2.65 ± 5.51	<0.0001	0.48 (moderate)
Antera 3D^®^					
Pigmentation	75.75 ± 27.72	61.89 ± 29.02	−12.14 ± 26.60	<0.01	−0.46 (moderate)
Vascularity	107.69 ± 19.55	92.00 ± 24.20	−13.43 ± 22.64	<0.001	−0.59 (moderate)
Volume	2.59 ± 1.46	1.90 ± 1.58	−0.65 ± 0.78	<0.0001	−0.83 (large)

**Table 3:** Baseline values of scar assessment parameters measured by VSS, dermoscopy, and Antera 3D^®^ in 35 patients (mean ± SD and coefficient of variation).

	Mean ± SD	CV (%)
VSS		
Pigmentation	1.77 ± 0.65	36.45%
Vascularity	1.60 ± 0.85	52.95%
Height	1.69 ± 0.80	47.22%
Pliability	2.37 ± 0.81	34.05%
Overall	7.46 ± 2.37	31.76%
Dermoscopy		
Green value	143.90 ± 17.37	12.07%
a*	35.58 ± 6.79	19.09%
L*	68.63 ± 11.45	16.68%
ANTERA 3D^®^		
Pigmentation	69.91 ± 30.35	43.41%
Vascularity	107.09 ± 18.34	17.13%
Volume	15.87 ± 14.48	91.21%

## Data Availability

The original contributions presented in this study are included in the article. Further inquiries can be directed to the corresponding authors.

## Supplementary Materials

The following supporting information can be downloaded at: https://www.mdpi.com/article/10.3390/biomedicines14040784/s1, Table S1. Comparison of VSS scores before and after treatment using Wilcoxon signed-rank test for 36 scar scars from 18 patients.

## Abbreviations

The following abbreviations are used in this manuscript:

PPS	pediatric pathological scars
3D	three-dimensional
VSS	Vancouver Scar Scale
SRMs	standardized response means
CV	coefficient of variation
IRB	Institutional Review Board
ROI	region of interest
SD	standard deviation
