# Supplementary material for: Comparison of Quantitative Evaluation and Conventional Scar Scale Analysis for Pediatric Pathological Scars
Source: Biomedicines. 2026 Mar 30;14(4):784. doi: 10.3390/biomedicines14040784 (PMC13113962; doi:10.3390/biomedicines14040784)
Supplement: Supplementary file 1 [file biomedicines-14-00784-s001.zip › biomedicines-4190124-supplementary.pdf]

Table S1. Comparison of VSS scores before and after treatment using Wilcoxon signed-rank test for 36 scar scars from 18 patients.

	p value (Wilcoxon signed rank test)
VSS	
Pigmentation	>0.05
Vascularity	>0.05
Height	<0.05
Pliability	<0.0001
Overall	<0.001
